# Shall Medical Societies Enforce Their Laws, Etc.

**Published:** 1871-04

**Authors:** 


### "SHALL MEDICAL SOCIETIES ENFORCE THEIR LAWS? SHALL THEY GOVERN OR BE DEFIED?"

The above caption may be suspected to be the language of the editors of the COMPANION. It is very similar to that we sometimes employ. It affords us pleasure, however, to say it comes from the pen of Prof. Gaillard, the talented editor of the Richmond and Louisville Medical Journal. In order to show the force of the above questions, we beg to refer to the circumstances calling them forth :

At the last meeting of the American Medical Association, a resolution was passed prohibiting its members, and those of local societies represented in it, from charging less than five dollars in examining parties making application for life insurance.

It appears that the College of Physicians and Surgeons of the city of Louisville, subsequent to the meeting of the American Medical Association, adopted the recommendation of that body upon the subject. The insurance companies of that city exerted every effort to defeat this action. It was brought to the knowledge of the society that one of its members—Dr. A. B. Cook—continued to make these examinations for three dollars. Charges were preferred against him, and he was summoned to appear before a committee duly appointed to investigate the matter. He declined to appear, alleging that the notice was not officially signed This having been corrected, he was notified again. He then declined to answer to the charges, claiming that he was not a member, for the reason that he had failed to attend its meetings, and had, during that time, paid no fees. It is shown, however, that he had presided at one of its meetings during the period specified. The society, after delaying the matter as long as possible, unanimously passed a resolution of expulsion, "and desired that the facts of such expulsion should be published in the newspapers and be made known to other societies, the State Medical Society and the American Medical Association." "Such are the facts," says the editor, "given with care and unquestionable justice."

Dr. Cook then appealed to the public through the newspapers, thus carrying his cause from a professional tribunal to that of the unprofessional public, invoking them to sustain his violation of medical law and casting reproach upon his professional brethren.

Dr. Gaillard remarks that "these facts are of local importance; their application is universal." Does not every local issue, involving right or wrong, have an universal application? Is principle less revered because the results arising from it are locally circumscribed? "When the principles involved are applicable to all societies, the matter ceases to be of local interest, and manifestly concerns every physician." We have before stated this principle and are glad to see it announced by so able an expounder of our law.

That medical organizations have the right to control their members, and the power to enforce their rules when the ethics are violated, admits of no discussion. If there be penalties attached to ethical violations, what are those penalties? Pray, why did the society of Louisville notify other societies and the National Association of the expulsion of Dr. Cook? Was it done to humiliate or injure in any way an expelled member? By no means! It was, to the members of that society, a most painful act. What, then, was the object? Was it intended as a recommendation—a passport—to all other professional gentlemen to receive Dr. Cook as a good and worthy brother? If so, why expell him? Why notify other societies of the fact, if he was still entitled to professional affiliation, as well *after* as *before* that notification? Is it possible that any would be so unmindful of their professional obligations as to forfeit their true position and hazard the reproach of their brethren so far as to voluntarily sustain a relationship to Dr. Cook, which no member in full fellowship with that or any other society could occupy, without being subject to the same penalty? Can any member of that society, or any other, recognize him? If not, then, pray what *unusual* privileges does any other professional man enjoy by right of which he can recognize him? If such an individual can rightfully claim to stand fair with organizations, and at the same time to recognize individuals expelled from their organizations, these organizations are a farce and their members dupes.

Was it ever intended that "go-betweens" should be so placed as to reap all the advantages of respectability conferred by organizations, and at the same time receive and enjoy the forbidden fruits—the pecuniary benefits of association and affiliation with outsiders—either those who have been expelled or those who, by irregular acts have never had any connection with them? If so, why have organizations at all, since observance of their laws fetter the hands of the good, and confers freer liberty, and greater benefits and privileges upon the supporters of violaters? If membership with medical societies presupposes no duties to be performed; confers no privileges to be enjoyed as the result of obedience to its laws; demands no obligations to be shared, then the sooner they are disbanded the better. If no distinct lines are to bedrawn between those who obey the law and those who violate it, or with those who recognize and affiliate with those who do, then organization is a trap and a delusion. If those who defend and sustain the law are to be ignored; if even the sentiment of approval and endorsement is withheld, because such endorsement may offend irregular and expelled members, then organizations are stupendous humbugs and its members triflers with principles and with laws! They become aiders and abetters of those declared to be unworthy, and pass adverse judgment upon those who maintain and support the very laws they were commanded and required to sustain.

A few carpers may say that the expulsion of Dr. Cook for violating the fee bill was the infliction of too grave a punishment for so slight an offense. But the smallness of the offense was not the reason; it was for the commission of a lawless act, for which he was expelled, an act by which he sought to take an undue advantage of his brethren. There are no minor and major laws when principle controlls. Principle does not stop to inquire as to degrees in obedience to, or violation of, ethical rule. Expulsion for the infraction of even the least law carries with it all the force and culpability of the greatest—to violate one is to be guilty of all. One expelled man, therefore, stands no fairer before the law than another ; all are aliens, beyond the reach of recognition or mercy, until the infliction of righteous penalties and genuine repentance shall work their reformation and restoration without injury to the body or any member of it. Such restoration, when it does occur, to be true and just, must not be effected to the damage of the organization, or to any who have conformed to law and principle. There must be an obliteration of the past, which shall in no wise taint the acts of the faithful for their firmness, or work disastrously to their interests.

Some may say—some do say in parallel cases—that "discourtesy" on the part of Dr. Cook was the cause of his expulsion, and weak reasoners, with a sophism peculiar to that class, pretend to convince themselves and others that some grades of expulstion works no forfeiture of position, recognition or affiliation.

True, his expulsion, in one sense, was for "discourtesy,"—but this was not all, and can never be all in any case of expulsion. He had violated the law, not only by his acts of "discourtesy," but other principles, moral as well as professional, of the ethics. There can be no "discourtesy" shown a body, worthy of excommunication, without other and graver violations of principles underlying "discourteous" action.

Such opinions of the power and influence of medical organizations are entertained by few. Such sentiments are not the upgushings from hearts moved by high motives and pure principles, but are conceived in malice or born of selfish and mercenary impulses. It is surely time for the profession to assert its principles in no mistakable language, and to convince the gainsaying of one truth at least—that no man, however learned, however bold, however vain of his own powers, shall be permitted to violate the principles of the profession with impunity, or affiliate with those who do, and at the same time hold his connection with it. In the language of Dr. Gaillard, we, too, affirm that "it may be pardoned when the uninformed, the carpers, the jesters, the harlequins, professional and secular, affect to deride the code of ethics ; but all physicians of good repute acknowledge a proper and just allegiance to it." "Unless medical societies are to become a contemptible farce, they must in all such cases manifest firmness and decision ; they must uphold their laws."

